# Hashimoto’s Encephalopathy: A Rare Cause of Seizure-like Activity

**DOI:** 10.7759/cureus.14626

**Published:** 2021-04-22

**Authors:** Heba Osman, Aaron Panicker, Paul Nguyen, Mira Mitry

**Affiliations:** 1 Department of Internal Medicine-Pediatrics, Wayne State University Detroit Medical Center, Detroit, USA; 2 Department of Internal Medicine, Wayne State University School of Medicine, Detroit, USA; 3 Department of Internal Medicine, Wayne State University Detroit Medical Center, Detroit, USA

**Keywords:** hashimoto’s, altered mental state, cognitive impairment and dementia, hashimoto’s encephalopathy, autoimmune encephalopathy, hashimoto's thyroiditis

## Abstract

Hashimoto’s encephalopathy is an uncommon disorder that may present with a wide variety of different neurological signs and symptoms that can include acute altered level of consciousness, psychosis, seizures, ataxia, dementia, myoclonus, and stupor. We present a case of a 60-year-old female patient who was admitted to the internal medicine floor for workup for seizures of unknown etiology. Investigations, including a complete blood count, basic metabolic panel, magnetic resonance imaging (MRI) of the brain, cerebrospinal fluid analysis, and NMDA (N-methyl-D-aspartate) receptor encephalitis screen, were all unremarkable. Thyroid-stimulating hormone levels and anti-thyroid peroxidase antibodies were found to be elevated, suggesting an underlying etiology of Hashimoto’s thyroiditis. Treatment with corticosteroids and levothyroxine can lead to resolution of symptoms. This case report is presented to suggest the importance of serological screening for anti-thyroid antibodies in the workup of all patients with unknown causes of encephalopathy along with providing a review of the literature.

## Introduction

Cognitive impairment and altered mental status is a frequent presentation in patients who come to the hospital [1]. Hashimoto’s encephalopathy (HE) is a rare immune-mediated disorder characterized by symptoms of acute encephalopathy associated with increased anti-thyroid antibody levels that usually improve with steroids. In fact, the condition is sometimes referred to as steroid-responsive encephalopathy associated with autoimmune thyroiditis (SREAT) [2]. It was first described in 1966 by Lord Brain [3]. Later, case reports and series were able to further describe the clinical findings of this disorder.

The pathophysiology of this disease has yet not been confirmed but has been presumed to be of autoimmune origin. The correlation of Hashimoto’s thyroiditis and HE is prevalent; however, the exact cause of encephalopathy has not yet been confirmed [4]. As with most rare conditions, the prevalence of the disorder is difficult to predict; however, it is estimated to have a prevalence of two in 100,000 [5]. Similar to many autoimmune diseases, it is more common in women than in men, with the mean age of onset being between 45 and 55 years [5]. Symptoms can include seizures, encephalopathy, myoclonus, dementia, pseudoseizures, and psychosis. Seizure disorders are found in approximately two-thirds of patients diagnosed with HE, and anticonvulsant therapy alone is usually not effective. It is a diagnosis of exclusion and is associated with increased anti-thyroid antibody and thyroid peroxidase antibody (TPOAb) levels [6].

## Case presentation

**Figure 1:**
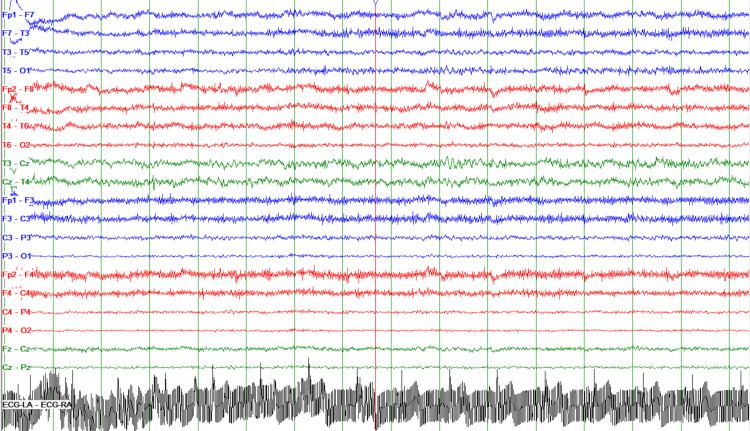
EEG recording demonstrating focal right temporal slowing

A 60-year-old female with a history of depression, chronic obstructive pulmonary disease, heart failure with preserved ejection fraction, hypertension, hyperlipidemia, and non-epileptiform seizure disorder presented to the emergency department (ED) for altered mental status after reports of seizure-like activity occurring at home. The patient was taking alprazolam at home but ran out of all her medications two weeks prior to admission. After arriving to the ED in a post-ictal state, she went into respiratory distress and required intubation. The patient was loaded with levetiracetam and admitted to the intensive care unit. Alprazolam was also started as it was thought that it may be likely that the patient’s episode was a result of benzodiazepine withdrawal. The patient was then extubated and transferred to the internal medicine floor for prolonged long-term electroencephalography (EEG) monitoring with video recording. The patient continued to have episodes of seizure-like activity while on levetiracetam that did not abate even when switched to phenytoin. These episodes were associated with chewing motions, head and body shaking, and rocking movements. During the majority of the seizures, the patient had low-amplitude generalized shaking episodes of the body with some rocking movements back and forth. All seizures appeared to be quite similar and lasted between 12 and 39 minutes. EEG revealed three episodes of clinical and electrographic seizures originating from the right hemisphere, maximal in the temporal region. These events were associated with prolonged and distinctive focal slowing in the right temporal region (Figure 1).

**Figure 2:**
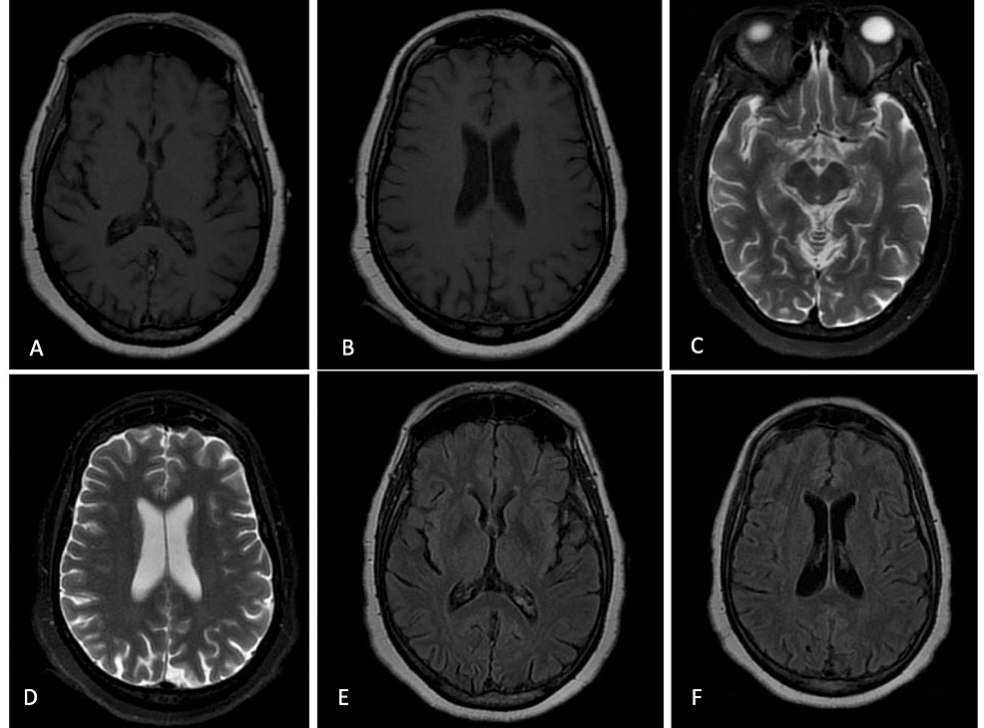
A 60-year-old female with Hashimoto's encephalopathy. Brain MRI was performed at the onset of symptoms. Axial T1 (A and B), axial T2 (C and D), and axial FLAIR (E and F) show no extra-axial fluid collection, intracranial mass, or mass effect. Contrast-enhanced images demonstrate no abnormal intracranial enhancement. All midline structures are completely formed and demonstrate normal morphology. No heterotopic gray matter or cortical abnormality is identified. The ventricular size and brain volume are proportional and age-appropriate. Essentially a negative examination. FLAIR, fluid-attenuated inversion recovery

Brain magnetic resonance imaging (MRI) was negative and did not reveal any abnormal intracranial enhancement (Figure 2).

Serum thyroid function test showed thyroid-stimulating hormone (TSH) of 10.00 mIU/L (reference intervals: 0.45-5.33 mIU/L), free thyroxine (FT4) of 0.76 ng/dL (reference intervals: 0.7-1.70 ng/dL), and anti-TPOAb of 310.9 IU/L (reference intervals: 0.20-9.0 IU/L). Our patient’s workup also included a negative NMDA (N-methyl-D-aspartate) receptor panel to rule out autoimmune encephalitis. The findings on EEG along with an elevated TPOAb were suggestive of HE, and thus treatment with steroids was initiated. Given the indications for HE, the patient was treated with oral methylprednisolone 500 mg twice a day for five days. Her symptoms improved significantly and neuropsychiatric symptoms fully resolved. Endocrine was also consulted and recommended that the patient start levothyroxine 50 mcg daily. The patient was discharged in a stable condition, and follow-up visits over the next few months did not reveal any recurrence in seizures.

## Discussion

The pathogenesis of HE remains poorly understood, and the disorder is often misdiagnosed. Concerns have even been raised regarding the commonly used poorly defined term, Hashimoto’s encephalopathy. The limited evidence on the pathogenicity of antithyroid antibodies suggests that HE is more likely to represent a group of disorders due to a variety of autoimmune causes [6].

The presentation can vary and often has a relapsing and remitting course [7]. In the more acute form, symptoms of confusion, hallucinations, epileptic seizures, tremor, myoclonus, focal neurologic abnormalities such as ataxia, and rapidly progressive dementia may be present [8]. Patients with the chronic form of HE may be presumed to have a form of dementia. These symptoms are seen in various neurological pathologies and elicit a large differential diagnosis. They may be vascular (multi-infarct dementia, cerebral amyloid angiopathy), infectious (neurosyphilis, HIV dementia), toxic-metabolic (Wernicke’s encephalopathy, B12 deficiency), autoimmune (HE, NMDA receptor encephalopathy), metastatic/neoplastic, or neurodegenerative (Creutzfeldt-Jakob’s disease, Alzheimer’s disease, frontotemporal dementia) in nature [8]. Thus, it is important to rule out other etiologies with a thorough history and examination along with obtaining a complete blood count, basic metabolic panel, blood cultures, urinalysis, cerebrospinal fluid (CSF) cultures, MRI, and EEG. The finding of elevated anti-TPOAb titers or antithyroglobulin antibody (TgAb) along with the exclusion of other causes of encephalopathy support the diagnosis of HE. Diagnostic criteria were put forth by Castillo et al., as outlined in Table 1 [2].

**Table 1:** Diagnostic criteria for Hashimoto’s encephalopathy utilized by Castillo et al. TSH, thyroid-stimulating hormone; CSF, cerebrospinal fluid

Diagnostic Criteria
Encephalopathy manifested by cognitive impairment and one or more of the following: neuropsychiatric features (hallucinations, delusions, or paranoia), myoclonus, generalized tonic-clonic or partial seizures, or focal neurologic deficits
Presence of serum thyroid antibody
Euthyroid status (TSH: 0.3-5.0 mIU/L) or mild hypothyroidism (TSH: 5.1-20.0 mIU/L) that would not account for encephalopathy
No evidence in blood, urine, or CSF analyses of an infectious, toxic, metabolic, or neoplastic process
No serologic evidence of autoantibodies to indicate another diagnosis
No findings on neuroimaging studies indicating vascular, neoplastic, or other structural lesions to explain the encephalopathy
Complete or near-complete return to the patient's neurologic baseline status with steroid treatment

Chaudhuri and Behan reported a series of 18 patients with HE with varying clinical presentation, with the most common presentations being coma, seizures, and focal neurological deficits [4]. Olmez et al. reported a series of 13 patients also with varying presentation. The most common presentations were stroke-like symptoms or seizures (seen in seven patients) and subacute progressive decline in cognitive function (seen in four patients) [9]. The patients all had variable thyroid status: eight patients were hypothyroid, three patients were euthyroid, and two were subclinical hypothyroid [9]. Though HE is a diagnosis of exclusion, it is important to note that the symptoms may occur in euthyroid, subclinically hypothyroid, and hyperthyroid patients [6,10].

Treatment of seizures with an anticonvulsant, such as phenytoin, may be necessary as a temporary measure. However, in some patients, seizures do not respond to anti-seizure drugs but may in fact respond to steroid therapy, such as in our patient [4]. Olmez et al. reported that steroid treatment resulted in rapid resolution of symptoms in 12 of the 13 patients [9]. In addition, neurological status does not appear to improve with thyroid therapy alone in patients with concurrent hypothyroidism [2]. Along with corticosteroid therapy, concurrent treatment of the underlying dysthyroidism showed to have a 92% improvement of symptoms [6]. Other forms of immunomodulation have also been utilized in patients with HE, which include azathioprine, methotrexate, and immunoglobulin [2].

## Conclusions

HE is rare and can present with a variety of clinical presentations. It should be considered as a differential for a patient presenting with unexplained cognitive impairment. It is crucial to detect and treat HE, as early treatment with steroid therapy has a good prognosis. A screening test of thyroid function test and TPOAb should be incorporated in the confusion screen for patients presenting with acute or subacute cognitive impairment. Other diagnoses should also be excluded with a full workup that includes labs, CSF studies, EEG, and imaging. Treatment with steroids should be considered, especially in patients with seizures that do not respond with anti-epileptics, along with the treatment of underlying thyroid disorder.
